# Left Atrial Appendage Occlusion and Post-procedural Antithrombotic Management

**DOI:** 10.3390/jcm13030803

**Published:** 2024-01-30

**Authors:** Anders Kramer, Giuseppe Patti, Jens Erik Nielsen-Kudsk, Sergio Berti, Kasper Korsholm

**Affiliations:** 1 Department of Cardiology, Aarhus University Hospital, Palle Juul-Jensens Boulevard 99, 8200 Aarhus, Denmark; anha@clin.au.dk (A.K.); je.nielsen.kudsk@gmail.com (J.E.N.-K.); 2 Department of Translational Medicine, University of Eastern Piedmont, 13100 Vercelli, Italy; giuseppe.patti@uniupo.it; 3 Department of Thoracic and Cardiovascular Diseases, Maggiore della Carità Hospital, 28100 Novara, Italy; 4 Department of Interventional and Diagnostic Cardiology, Fondazione CNR-Regione Toscana G, Monasterio, Ospedale del Cuore, 54100 Massa, Italy; sergio.berti@me.com

**Keywords:** left atrial appendage occlusion, ischemic stroke, anticoagulation, antiplatelet, atrial fibrillation

## Abstract

Left atrial appendage occlusion (LAAO) is an established alternative to oral anticoagulation for stroke prevention in atrial fibrillation. Antithrombotic therapy is used in the post-procedural period to prevent device-related thrombosis (DRT). The risk of DRT is considered highest in the first 45–90 days after device implantation, based on animal studies of the device healing process. Clinically applied antithrombotic regimens vary greatly across studies, continents, and centers. This article gives an overview of the evidence behind current antithrombotic regimens, ongoing randomized trials, and future post-procedural management.

## 1. Introduction

Atrial fibrillation (AF) is the most common cardiac arrhythmia, with a projected major rise in prevalence in the coming decades in consequence of the aging demographics [1,2,3]. The associated risk of ischemic stroke warrants life-long anticoagulation therapy, which is considered the mainstay stroke prevention therapy in AF [4,5]. However, percutaneous left atrial appendage occlusion (LAAO) is now an established alternative for stroke prevention, particularly among patients with contraindication to long-term oral anticoagulation (OAC) [4,5,6,7,8]. 

The LAAO procedure has undergone significant advantages since the pivotal PROTECT-AF trial documented its non-inferiority compared to vitamin K antagonists (VKA). Although subsequent studies have documented a decline in procedure- and device-related adverse events, device-related thrombosis remains an issue to be resolved. Current consensus recommends post-procedural antithrombotic therapy to reduce the risk of device-related thrombosis (DRT) and its associated risk of ischemic stroke [9,10,11,12]. The pivotal PROTECT-AF and PREVAIL trials applied a combined regimen of VKA and antiplatelet therapy (APT), yet real-world LAAO patients most often have a history of serious bleeding complications, with a high proportion even considered intolerant to OAC [13,14,15,16]. Consequently, the optimal antithrombotic strategy remains debatable as thromboembolic risk should be balanced against the bleeding risk, with major bleeding currently representing the most frequent adverse event after LAAO [17,18,19,20,21]. 

In this review, we describe the therapeutic rationale behind post-LAAO antithrombotic treatment, and provide an overview of current evidence supporting the efficacy and safety of various antithrombotic strategies. Lastly, we provide an overview of ongoing studies comparing different post-procedural regimens and discuss future directions. 

## 2. Therapeutic Rationale

The primary justification for post-LAAO antithrombotic therapy is to prevent the formation of DRT on the atrial device surface during the endothelization phase. While the LAAO procedure itself has developed to a point where it may be considered low risk, the rates of DRT have remained unchanged at 2–5% across most trials, devices, and populations [22,23]. 

### 2.1. Hemostatic Changes 

**Figure 1:**
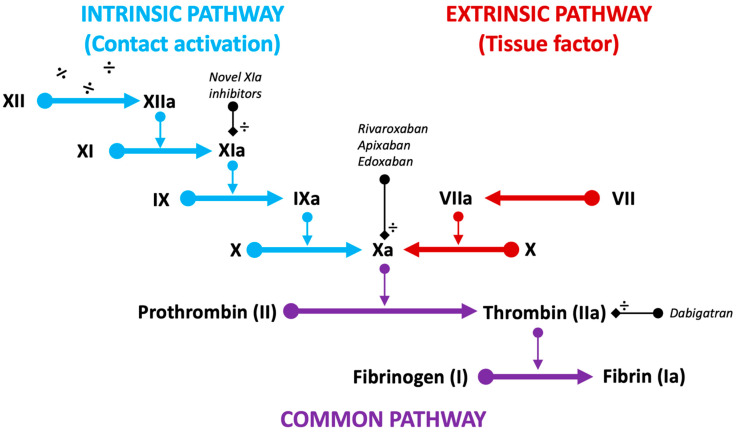
Schematic representation of the intrinsic (**blue**), extrinsic (**red**), and common (**purple**) pathways of the coagulation cascade. Target factors of current DOACs (Xa and IIa) and novel factor inhibitors (XIa) are displayed (**black**).

Experimental canine studies indicate complete endothelialization of the device surface to occur within 45–90 days [13,24,25,26]. During this period, the negatively charged device surface remains in contact with the circulating blood, promoting adsorption of plasma proteins, platelet adhesion, and contact activation of the intrinsic coagulation pathway (Figure 1) [27]. 

The specific biochemical impact of LAAO on coagulation and platelet activity has been investigated in two smaller studies [28]. These data indicated a significant increase in plasma levels of prothrombin factor 1 + 2 (F1 + 2) and thrombin-antithrombin complex (TAT), both byproducts of prothrombin to thrombin conversion. This increase was immediate, with a subsequent decline towards baseline levels around one month post-procedure [29,30]. The increase was less pronounced among patients on direct oral anticoagulation (DOAC), as observed in the randomized ADRIFT study [31]. 

Rodes-Cabau et al. investigated platelet activation through changes in P-selectin and CD40 ligand plasma concentrations, and found no significant elevation of platelet reactivity [30]. 

Based on a substantial reduction in hemostatic activation after the first post-procedural month, these biochemical findings seem to support the timing of endothelialization observed in preclinical canine studies [13,24,25,26]. Additionally, they suggest contact activation of the coagulation system as the potential primary driver of thrombus formation following LAAO. This would suggest DOACs, as opposed to antiplatelets, to prevent DRT; however, most patients in these non-randomized biochemical studies were actively treated with dual antiplatelet therapy (DAPT) or single antiplatelet therapy (SAPT) at the time of blood sampling. This likely reduces the measurable platelet response. The HEMO-LAAO (ClinicalTrials.gov: NCT05011981) and POPULAR-LAAO (ClinicalTrials.gov: NCT04705688) studies are currently ongoing and scheduled to further investigate the initial hemostatic findings with extended analyses of both coagulation and platelet activation following LAAO. 

### 2.2. Additional Risk Factors

Besides hemostatic changes, studies have identified various patient and procedural factors associated with the occurrence of DRT (Table 1). 

Some are modifiable risk factors, such as deep device implantation, which may represent an important target for minimizing the risk of DRT [35,38,41,42,43]. Furthermore, several non-modifiable patient factors have been associated with increased DRT risk, like CHA_2_DS_2_-VASc score, age, female sex, prior stroke, non-paroxysmal AF, and reduced left ventricular ejection fraction (Table 1) [11,37,38,42]. Across studies, it appears that factors affecting flow conditions, as well as hypercoagulability disorders, appear to impact the risk, in accordance with the triad of Virchow [32,35,38]. These factors should prompt further therapeutic considerations, guiding optimization of the discharge antithrombotic regimen in the individual patient, while the bleeding risk warrants consideration. Here, the strongest predictors appear to be prior major bleeding, age, renal failure, and presence of anemia, which are all highly prevalent among LAAO-recipients [44,45]. 

## 3. Antithrombotic Strategies 

Antithrombotic regimens have varied greatly across studies, continents, and centers (Table 2, Table 3 and Table 4). Available supporting evidence is primarily based on non-randomized data or extrapolations from other fields of interventional cardiology. 

**Figure 2:**
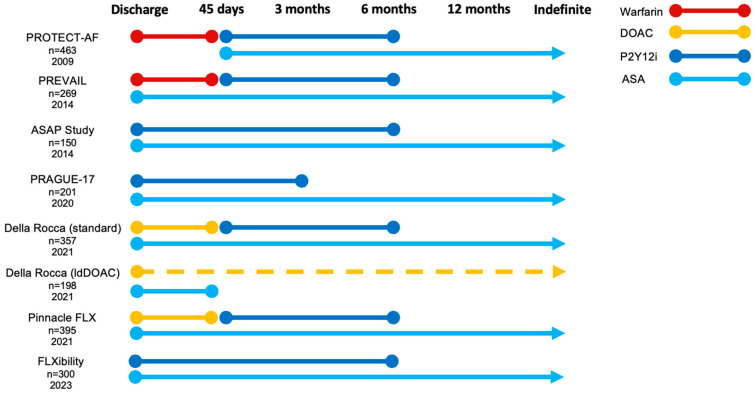
Recommended post-procedural antithrombotic strategies across large LAAO trials and registries. ASA, acetylsalicylic acid; DAPT, dual antiplatelet therapy; DOAC, direct oral anticoagulation; ldDOAC (dotted line), low-dose direct oral anticoagulation; P2Y12i, P2Y12 inhibitor.

In the US, the approved post-procedural antithrombotic approach was initially a short period of VKA therapy combined with acetylsalicylic acid (ASA) before conversion to DAPT, reflecting the PROTECT-AF and PREVAIL trial regimen (Figure 2) [14,15]. 

This approach was partially derived from the complexity of designing these early trials, including safety considerations when defining an appropriate control population, and the trials focused on efficacy to prevent stroke, and the fact that the trials included patients otherwise eligible for anticoagulation [16]. Consequently, FDA approval required LAAO candidates to be suitable for short-term VKA treatment. However, real-world data from the extensive US post-market NCDR registry (*n* = 31,994) show that only 37% of Watchman-implanted patients from 2016–2018 received the FDA-approved post-procedural regimen of VKA and ASA, which was mainly replaced by DOACs [51]. 

Meanwhile, in Europe, an antiplatelet-focused approach to post-LAAO therapy has dominated [47]. In part, this difference might be explained by the early European advancement of the Amplatzer LAAO devices, recommending DAPT at discharge, as well as European LAAO candidates being at higher bleeding risk and considered contraindicated to even short-term OAC. Accordingly, a large amount of single- and multicenter registries display the European experience with APT (Table 2 and Table 4) [12,38,46,47,52,53]. Recently, DAPT was approved in the US for post-Watchman antithrombotic treatment. The Amulet device has been FDA-approved with a DAPT discharge regimen, and several studies on post-procedural DOAC-treatment are ongoing in both the US and Europe.

The present comparative evidence relies on an indirect comparison of treatment strategies across observational studies, which may be difficult due to cohort heterogeneity, high heterogeneity in type and duration of antithrombotic treatment due to variabilities in practice patterns among physicians, and potential variations in the applied study endpoint definitions. While stroke represents a hard endpoint throughout studies, major bleeding rates may be harder to compare (Table 2). 

Similarly, DRT represents a difficult diagnosis, with some studies indicating disagreement between reviewers in one-third of cases [54]. 

### 3.1. Oral Anticoagulation

#### 3.1.1. Vitamin K Antagonists

VKA in combination with ASA was initially mandated in both the PROTECT-AF and PREVAIL trials as well as their continued access registries, CAP and CAP2, respectively. The later introduction of DOACs resulted in a temporal transition away from the FDA-approved VKA regimen. In an NCDR LAAO registry analysis, the risk of adverse events appeared less with a DOAC-only approach, compared to both VKA plus aspirin and DOAC plus aspirin. The difference was primarily driven by less bleeding, without a significant difference in ischemic events [51]. 

A meta-analysis of the PROTECT-AF, PREVAIL, CAP, and CAP2 cohorts provided systematically collected data on 1877 patients treated with VKA and aspirin after Watchman implantation [6,55]. In this collective cohort, TEE-verified DRT was present in 3.7% of implanted patients during 12 months follow-up and was associated with an increased risk of thromboembolic events (RR 3.6 [95%CI 2.2; 5.8]) [11]. Assessing the CAP registries, CHA_2_DS_2_-VASc and HAS-BLED scores were higher compared to the respective trials, potentially increasing translatability towards the current real-world LAAO population representing high-risk patients. The ischemic stroke rates were 1.3/100 patient-years and 2.2/100 patient-years in CAP and CAP2, respectively [56]. The major bleeding rate in the CAP registry was 3.05/100 patient-years [56]. In the NCDR LAAO registry, including almost 32.000 patients, major bleeding rates at six months was approximately 3.8% in VKA-only (7.1/100 patient-years), and 5.0% (7.9/100 patient-years) among those discharged on the recommended VKA + ASA regimen (Table 2) [51]. The bleeding rate was comparable to patients discharged on DOAC or DAPT with 3.7% (392/10,597) and 3.3% (49/1614), respectively. Similar findings have been reported for other cardiac interventions, like the POPular-TAVI trial (*n* = 313), displaying increased rates of non-procedural bleeding among patients on VKA + Clopidogrel compared to VKA alone post-procedurally [57]. 

Ischemic stroke rates at six months appeared overall low and comparable among various antithrombotic regimens in the NCDR registry [51]. Non-randomized data comparing VKA and DOAC discharge have suggested both to be equally safe and effective [48]. Nevertheless, most studies recorded only short-term follow-up, with a low absolute number of events and a high risk of selection bias. Conclusively, the combination of VKA and platelet inhibitors appears to increase bleeding risk without substantial impact on the thromboembolic risk. 

**Table 3:** Overview of studies reporting specifically on outcomes using an oral anticoagulation discharge regimen.

Anticoagulation-Specific Studies						DRT *		Ischemic Stroke **		Major Bleeding **		CV Mortality **	
Study	Publ.	n	Device(s)	Random	FU (Months)	DOAC	VKA	DOAC	VKA	DOAC	VKA	DOAC	VKA
**VKA**													
PROTECT-AF [14]	2009	463	WM	Yes	18 (±10)				2.2%		3.5%		0.7%
PREVAIL [15]	2014	269	WM	Yes	18				1.9%				2.6%
**DOAC**													
Della-Rocca (DOAC) [58]	2021	357	WM	No	14 (IQR; 12, 15)	3.4%		1.7%		3.4%		2.8%	
Della-Rocca (ldDOAC) [58]	2021	198	WM	No	13 (IQR; 12, 14)	0.0%		0.0%		0.5%		2.0%	
Pinnacle FLX ^ [20]	2021	395	WM FLX	No	12	1.8%		2.6%		7.9%		4.4%	

* DRT rates are estimated based on patients with available follow-up imaging. ** Annual rates were chosen where possible for presentation of clinical events. ^ Strong recommendation towards specific AT regimen. Studies randomizing between LAAO and pharmacotherapy as well as between different AT regimens are marked as “random”. AT, antithrombotic therapy; DOAC, direct oral anticoagulation; DRT, device-related thrombosis; LAAO, left atrial appendage occlusion; ldDOAC, low-dose direct oral anticoagulation; VKA, vitamin K antagonist; WM (FLX), Watchman (flx).

#### 3.1.2. Direct Oral Anticoagulation

The safety benefit of DOACs versus VKA has been established in the general AF population [59]. However, a potential caution of a DOAC-based approach after structural heart interventions was raised after discouraging results from the randomized GALILEO trial on transcatheter aortic valve implantation [60,61]. Here, subclinical leaflet thrombosis was reduced on CT follow-up, yet thromboembolic and bleeding risk were increased among low-dose rivaroxaban-treated patients. The RE-ALIGN and PROACT Xa trials, investigating DOAC with mechanical valve prosthesis, were prematurely terminated due to both increased thromboembolic and bleeding risk among DOAC-treated patients [60,61,62]. Additionally, the recently published FRAIL-AF was terminated early due to harm, converting frail elderly AF patients from VKA to DOAC therapy, a population holding a large representation among real-world LAAO patients [63]. 

Nevertheless, the Pinnacle-FLX trial appeared to affirm the safety of a DOAC-based post-procedural strategy following implantation of the Watchman FLX (Table 3, Figure 2) [20,59,64,65,66,67]. The primary safety and efficacy endpoints were focused on the procedure itself, yet annual rates of DRT, ischemic stroke, and major bleeding were reported to be 1.8%, 2.6%, and 7.9%, respectively. The NCDR registry analysis of post-procedural antithrombotic therapy also confirmed a low and comparable risk of ischemic events with DOAC compared to both VKA and DAPT [51,68].

Following these results, the use of a low-dose DOAC (primarily Apixaban) has been investigated, with promising results. Della-Rocca et al. reported a significant reduction in the composite endpoint of DRT, thromboembolic events, and major bleeding compared to full-dose DOAC after a median follow-up of 13 months [58]. No cases of DRT or ischemic stroke were observed in the low-dose DOAC group, and major non-procedural bleeding was only 0.5%. In the full-dose DOAC group, non-procedural bleeding occurred in 3.4%, while, both DRT and ischemic stroke occurred in 3.4% and 1.7% of patients. The restricted sample size, risk of selection bias, and confounding by indication in this non-randomized comparison need to be highlighted, although the cohorts appeared comparable concerning CHA_2_DS_2_-VASc score, thromboembolic history, and a priori risk of bleeding. The study findings are, however, supported by biochemical results from the randomized ADRIFT pilot study, as well as the experiences from another small non-randomized study, underlining the need for dedicated randomized trials (Table 2 and Table 3) [31,69].

The randomized ADALA trial (ClinicalTrials.gov: NCT05632445) recently presented results of a pre-planned interim analysis (*n* = 90) at the 2023 EuroPCR congress. Low-dose DOAC was associated with a lower risk of DRT and bleeding compared with DAPT following primarily Amulet (68%) or Watchman (23%) LAAO. While these results suggest a potential safety advantage of low-dose DOAC, they need confirmation in larger cohorts as the study was significantly underpowered as the difference was driven by relatively few events in absolute numbers.

### 3.2. Antiplatelet Therapy 

DAPT has long been utilized as the primary antithrombotic strategy following LAAO with the Amplatzer Cardiac Plug (ACP) and Amulet and was recently FDA approved after Watchman FLX implantation in the US. The strategy was mainly derived from the experiences with atrial septal defect and patent foramen ovale closure with other Amplatzer devices, and meets the need for an OAC alternative, especially in the largely OAC-intolerant European LAAO-cohorts. 

In 2013, the ASA Plavix Feasibility Study (ASAP) investigated six months of DAPT with ASA and a P2Y12-inhibitor (P2Y12i) in 142 Warfarin-ineligible patients successfully implanted with the Watchman device (Figure 1) [70]. The annual risk of ischemic stroke was 1.7% and the DRTrate was 4.2%, considered comparable to the PROTECT-AF trial cohort (Table 4). In part, these results were reproduced in the EWOLUTION registry and the FLXibility study [21,47]. The FLXibility endpoints mirror those seen in both Pinnacle FLX and the EWOLUTION registry, suggesting equal effects of APT or OAC after Watchman FLX (Table 2, Table 3 and Table 4) [21].

Data for the Amplatzer devices are mainly derived from the two large ACP and Amulet registries, comprising more than 2000 patients [12,46]. The ACP registry included 50.7% of patients discharged on either DAPT (15.7%) or SAPT (34.7%), while in the Amulet Registry 57.7% were on DAPT and 22.4% on SAPT. The rates of DRT and ischemic stroke were comparable to other trials and registries, although major bleeding rates (BARC ≥ 3) for both DAPT-, SAPT-, and OAC-treated patients appeared higher, with many bleeds (29%) occurring within one week of the procedure (Table 2) [49]. Interestingly, the Amulet-IDE trial, comparing the Amulet device to the Watchman 2.5 device, reported comparable major bleeding and ischemic stroke rates, despite a DAPT approach being applied in the Amulet-group and a VKA plus/minus APT regime in the Watchman 2.5 group [18]. 

A minimalist approach with SAPT has also been investigated in high-risk patients. Results indicate a reduction in major bleeding risk while possibly maintaining efficacy [53,71]. The Amulet registry reported comparable DRT rates between SAPT- and DAPT-treated patients, with a potential reduction in bleeding (Table 2). Similar findings have been reported in smaller observational studies [50,72]. Nevertheless, this warrants confirmation in a randomized setting due to inherent risks of bias, particularly as some studies have suggested an association between SAPT or no discharge therapy and increased risk of DRT [43]. In the setting of TAVI, the randomized POPular-TAVI trial (*n* = 665) affirmed that SAPT therapy was associated with a lower risk of bleeding compared with DAPT (RR 0.6 [95%CI 0.4; 0.8]) [73]. Nevertheless, randomized data on efficacy of post-LAAO antiplatelet therapies are warranted.

**Table 4:** Overview of studies reporting specifically on outcomes using an antiplatelet discharge regimen.

Antiplatelet-Specific Studies						DRT *		Ischemic Stroke **		Major Bleeding **		CV Mortality **	
Study	Publ.	n	Device(s)	Random	FU (Months)	SAPT	DAPT	SAPT	DAPT	SAPT	DAPT	SAPT	DAPT
**DAPT**													
ASAP study [70]	2013	150	WM	No	14.4 (±8.6)		4.2%		1.7%				2.1%
Urena et al. [74]	2013	52	ACP	No	20.0 (±5.0)	0.0%		1.9%		1.9%		1.9%	
Weise et al. [52]	2016	298	ACP/AM/WM/WC	No	26.9 (±17.9)		2.6%		1.7%		3.9%		
Pracon et al. [38] ^	2018	99	ACP/AM/WM	No	12		7.1%		1.0%				6.1%
PRAGUE-17 [75] ^	2020	201	AM/WM (FLX)	Yes	19 (IQR; 12, 28)		3.4%		2.6%		3.8%		3.2%
FLXibility ^ [21]	2023	300	WM FLX	No	12		2.4%		2.0%		8.5%		5.1%
**SAPT**													
Rodriguez-Gabella et al. [71]	2016	31	ACP/AM/WM	No	19 (IQR; 12, 24)	3.3%		0.0%		3.2%		3.2%	
Korsholm et al. [53]	2017	107	ACP/AM	No	28 (IQR; 19, 38)	1.9%		2.3%		3.8%			

* DRT rates are estimated based on patients with available follow-up imaging. ** Annual rates were chosen where possible for presentation of clinical events. ^ Strong recommendation towards specific AT regimen. Studies randomizing between LAAO and pharmacotherapy as well as between different AT regimens are marked as “random”. ACP, Amplatzer cardiac plug; AM, Amplatzer Amulet; AT, antithrombotic therapy; DAPT, dual antiplatelet therapy; DRT, device-related thrombosis; LAAO, left atrial appendage occlusion; SAPT, single antiplatelet therapy; WC, WaveCrest; WM (FLX), Watchman (flx).

### 3.3. No Therapy 

A strategy without any post-procedural antithrombotics may be particularly desirable in LAAO recipients at high bleeding risk. While no studies exist reporting its systematic use, a few studies have reported event rates among selected patients discharged without any antithrombotic therapy (Supplementary Table S1). In the Amulet and the EWOLUTION registries, 23 (2%) and 65 (6%) patients were discharged with no therapy [12,47]. The absence of post-procedural therapy did not predict DRT formation, but the low number of patients and a selection of high-bleeding-risk patients with lower thrombotic risk may affect the outcome. Another study by Darmon et al. reported no incidents of bleeding, ischemic stroke, or DRT during 16 months follow-up in 22 patients discharged without therapy due to angiodysplasia (*n* = 12, 54.5%), intracranial bleeding (*n* = 12, 54.5%), mobility, or cognitive disorders (*n* = 10, 45.0%), or repeated bleeding on OAC (*n* = 9, 40.1%) [76]. As can be seen from these numbers, most patients held more than one of these risk factors. The above-mentioned studies are likely subject to heavy confounding by indication and selection bias, evident by a high a priori risk of bleeding and the very low number of the total cohort discharged without antithrombotic therapy (2–15%). Future device iterations incorporating membrane coating, altered surface properties, or reduced exposure of foreign material, like the current Watchman FLX Pro, Conformal, and Laminar devices, may spur further interest in more minimalistic post-procedural pharmacological approaches in the future.

### 3.4. Comparing Strategies

The ADRIFT trial, published in 2020, remains the only published randomized data on post-procedural therapy, utilizing a surrogate biochemical endpoint suggestive of thrombin generation [31]. Few metanalyses have been performed incorporating data from registries and randomized studies comparing LAAO to pharmacological stroke prevention. Li et al. included 32 studies (*n* = 12,326) in a meta-analysis comparing an OAC-based strategy to APT following LAAO [77]. Information was primarily derived from observational studies and a large propensity-score matched analysis of early Watchman trials [78]. Recently, Carvalho et al. performed a network meta-analysis of 41 studies (*n* = 12,451), comparing the association between a range of antithrombotic regimens and the occurrence of DRT, ischemic events, bleeding, and mortality [79]. The analyses found that no antithrombotic therapy after LAAO was associated with an increased risk of DRT compared to DAPT, DOAC (+/− APT), and VKA strategies. DOAC appeared with the lowest risk of thromboembolic and major bleeding events, while SAPT was associated with a higher thromboembolic risk compared with DAPT without any difference in bleeding risk. Though vulnerable to confounding from patient selection and a difference in devices used across the included studies, these meta-data offer a welcome outline of the existing evidence in the absence of randomized trials. 

## 4. Duration of Treatment

Besides the composition of post-procedural antithrombotic therapy, the optimal timing of de-escalation or complete discontinuation of post-procedural therapy remains another central question without much data to support current practice. A high proportion of DRT events are reported after de-escalation of (D)OAC therapy, questioning the timing of de-escalation [20,58]. Conversion from OAC to APT, downgrading from DAPT to SAPT, or completely terminating therapy varies greatly across studies, and is likely highly dependable on patient history, as assessed by the implanting physician. In the Amulet registry, 14.5% were completely off antithrombotic treatment at six months follow-up, reflecting a real-world practice of discontinuation. In the ACP registry, those de-escalating to SAPT or no therapy at last follow-up had reduced thromboembolic and bleeding risk [46]. 

Understanding device-healing patterns and potentially improving our post-procedural imaging follow-up algorithms to better predict the risk of adverse events and guide de-escalation of antithrombotic therapy are important areas to help mitigate the risk of DRT, while reducing the risk of bleeding events. 

### 4.1. Device Healing–In Vivo

In theory, the case for an initially more aggressive antithrombotic strategy rests on the timing of post-implantation device neo-endothelialization. Limited animal evidence guided the timing of de-escalation in the pivotal trials, and very little human confirmation exists to this day (Table 5).

Based on early canine studies, neo-endothelialization of the atrial device surface is a time-dependent process. Canine studies representing a total of 25 animals have been conducted on the first generation ACP (*n* = 13) and Watchman (*n* = 12) devices [13,24,25]. In summary, these studies showed complete coverage of both device types at 90 days, with Watchman devices displaying coverage of all exposed surfaces already at 45 days. While one study by Bass employed only a SAPT antithrombotic regimen in 10 ACP-implanted canines, the remaining two studies utilized the VKA and SAPT approach later adopted by the pivotal trials. Recently, data were published on the later generation Watchman devices (Watchman FLX and FLX Pro). In one study, utilizing a DAPT post-procedural therapy, complete endothelial coverage of the atrial surface of the Watchman FLX device was observed in four out of five cases at 45 days [26]. Comparably, in a later study using no antithrombotic therapy, at 45 days neo-endocardial coverage was observed in only two out of six Watchman FLX-implanted canines versus all cases implanted with the new Watchman FLX Pro [80]. In this same study, 90 day evaluation by scanning electron microscopy of FLX and FLX Pro devices, implanted in a porcine right atrial appendage model, displayed 62.8% and 87.7% endothelial coverage on the FLX and FLX Pro cases, respectively.

In vivo gross evaluation of human device healing is sparsely described in autopsy reports and from surgically removed devices (Table 5). A total of ten patient cases have been reported, all displaying incomplete neo-endothelialization at eight months to three years follow-up [81,82,83,84,85,86,87]. Among these cases, only two were reported to be below 70 years of age at the time of evaluation, potentially explaining, in part, the discrepancy compared with healthy young study animals [82,85].

**Table 5:** Overview of studies and case reports on in vivo device healing.

**Canine Studies**					
**Study**	**Publ.**	**n**	**Device Type**	**Antithrombotic Therapy**	**Results**
Bass [25]	2010	10	ACP	ASA	90 days: Atrial device surface covered by stable neointima. All animals displayed complete occlusion at both 30 and 90 days follow-up, as assessed by TEE and angiography.
Schwartz et al. [13]	2010	9	WM	VKA + ASA	3 days (*n* = 3): Atrial device surface covered by organizing thrombus. 45 days (*n* = 3): Thin white pannus across the atrial device surface. Endocardial ingrowth covering all exposed surfaces and in continuation with LA surface. 90 days (*n* = 3): A monolayer of endothelial cells covering healthy neo-endocardium.
Kar et al. [24]	2014	6	ACP (3) WM (3)	VKA + ASA	28 days: Complete neo-endocardial coverage of WM. Incomplete coverage of ACP disc. Remaining mild peri-device flow on TEE in both WM and ACP cases.
Kramer et al. [26]	2022	5	WM FLX	ASA + Clopidogrel	45 days: Thin layer of endothelial cells covering the device surface in four out of five cases. One case displayed only partial neo-endothelial coverage.
Saliba et al. [80]	2023	24	WM FLX (12) FLX Pro (12)	None	3 days (*n* = 6): Acute inflammation around fabric knots in 5.0% and 33.7% of FLX Pro and FLX cases, respectively. Reduced thrombus thickness in FLX Pro (0.3 mm) vs. FLX (1.5 mm) cases. 14 days (*n* = 6): Comparable inflammation in the two devices. Less thrombus thickness in FLX Pro (1.2 mm) vs. FLX (4.1 mm)—consistent with TEE findings. 45 days (*n* = 12): Smooth neo-endocardial coverage of 6/6 FLX Pro and 2/6 FLX devices.
**Porcine Studies**					
**Study**	**Publ.**	**n**	**Device Type**	**Antithrombotic Therapy**	**Results**
Saliba et al. [80]	2023	8	WM FLX (4) FLX Pro (4)	None	90 days: Nearly 100% white glistening tissue coverage across atrial surface of both device types. Endothelial coverage of 87.7% and 68.2% was seen across FLX Pro and FLX devices, respectively.
**Human Cases**					
**Study**	**Publ.**	**n**	**Device Type**	**Antithrombotic Therapy**	**Results**
Massarenti et al. [81]	2012	1	WM	VKA + ASA	10 months: No significant endothelialization observed. Fibrous connective tissue but no thrombus or neoplastic formation on pathology. Surgery.
Schiettekatte et al. [82]	2014	1	ACP	DAPT	1.5 years: Small thrombus associated with areas of incomplete endothelialization. Surgery.
Prosperi-Porta et al. [83]	2018	1	WM	DAPT	1 year: Well-seated but poorly endothelialized surface with associated device thrombus. Post-mortem.
McIvor et al. [84]	2019	1	WM	DOAC	3 years: Only limited superior endothelium across the atrial device surface. Device not adherent to the atrial wall. Surgery.
Sharma et al. [85]	2019	2	WM	VKA + ASA	1.5 years (Case 1): Complete lack of endothelialization. Surgery. 2 years (Case 2): Partial and incomplete endothelization. Surgery.
Ellis et al. [86]	2022	2	AM WM	-	2 years (Amulet): Endocardial growth across 60–75% of disc surface. Surgery. 8 months (Watchman): Endothelialization across 40–55% of atrial device surface. Post-mortem.
Vukomanovic et al. [87]	2022	2	WM	VKA + ASA	3 years (Case 1): Large mobile thrombus and non-endothelialized central screw hub. Surgery. 2 years (Case 2): Large superior thrombus on device surface. Non-endothelialized central screw hub. Surgery.

ACP, Amplatzer cardiac plug; AM, Amplatzer Amulet; ASA, acetylsalicylic acid; DAPT, dual antiplatelet therapy; DOAC, direct oral anticoagulation; VKA, vitamin K antagonist; WM (FLX), Watchman (flx); FLX Pro, Watchman FLX Pro.

### 4.2. Device Healing—Imaging

Traditionally, follow-up imaging after LAAO has been performed using transesophageal echocardiography (TEE) for both residual leak evaluation and DRT detection [54]. However, in recent years, postprocedural cardiac computed tomography (CT) has become an increasingly utilized non-invasive alternative. With its superior spatial resolution and contrast imaging, postprocedural CT has displayed an increased sensitivity in detecting both residual leaks and DRT [41,88]. Meanwhile, it allows for a thorough evaluation of the atrial device surface, including the evaluation of hypoattenuated thickening (HAT) as a marker of contrast sparring. Several studies have suggested low-grade HAT as a proxy for benign endothelialization, while high-grade HAT has been associated with a higher thromboembolic risk [26,41,89,90,91]. In one study, simultaneous CT and histology were performed in five canines, showing low-grade HAT to be associated with both macroscopic and histological evidence of neo-endothelialization [26]. Throughout several studies, HAT across the device surface on early follow-up CT has been observed in up to 61% and 64% of patients specifically implanted with Watchman FLX, while another study specifically evaluating ACP- and Amulet-implanted patients found HAT only in 6% of patients [26,41,91]. While this variability might suggest large differences in early endothelialization, the coupling of HAT with endothelial coverage needs additional validation. Visible contrast patency in the distal LAA with or without visible leak has also been suggested as a proxy measure of incomplete endothelial cover, in theory promoting an inverse relationship between low-grade HAT and LAA patency [92,93]. With both LAA patency and HAT being highly variable across studies, further investigation is needed to confirm this correlation and their association with benign healing. An improved understanding of this relationship could help guide the optimal timing of post-procedural antithrombotic therapy.

## 5. Discussion and Future Directions

Limited high-quality data exist to guide current post-procedural antithrombotic treatment. Accordingly, several studies are currently enrolling to enhance our understanding of post-procedural thrombus formation, device healing, and pharmacotherapy. Moreover, technological progress is continuously providing new iterations of existing device designs, as well as developing completely new device concepts. The present evidence appears to support either a DOAC-based approach without any APT, or an antiplatelet approach, where both DAPT and SAPT are equally used throughout studies. These three regimens are currently being tested in ongoing trials, but a secondary and equally important question relates to the duration of therapy.

### 5.1. Ongoing Trials 

**Figure 3:**
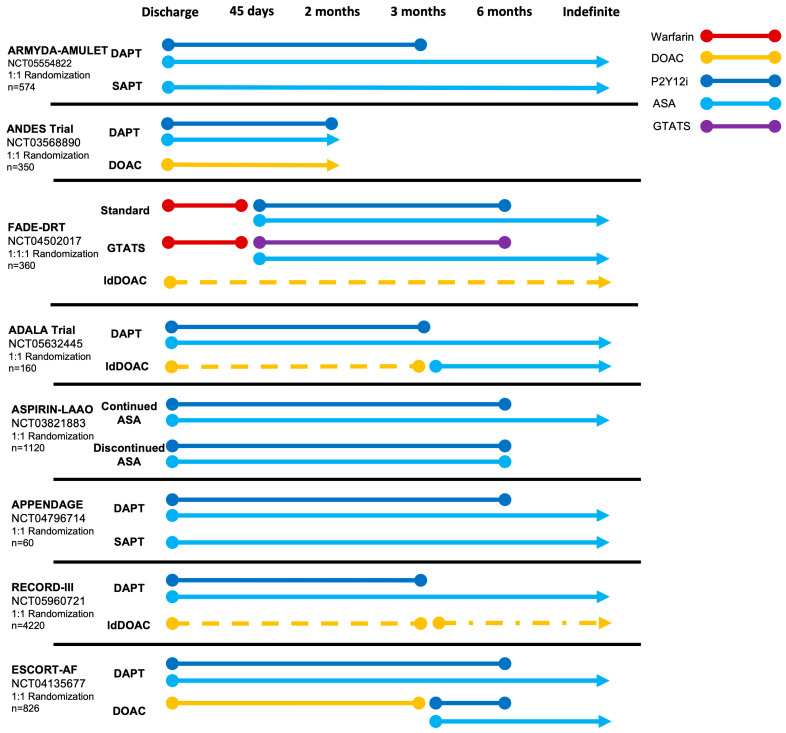
Ongoing randomized trials comparing post-procedural antithrombotic strategies after LAAO. ASA, acetylsalicylic acid; DAPT, dual antiplatelet therapy; DOAC, direct oral anticoagulation; GTATS, genetically tailored antithrombotic strategy; ldDOAC (dotted line), low-dose direct oral anticoagulation; P2Y12i, P2Y12 inhibitor.

The HEMO- (ClinicalTrials.gov: NCT05011981) and POPULAR-LAAO (ClinicalTrials.gov: NCT04705688) trials, will provide insights into the hemostatic changes following LAAO, potentially providing an evidence-based rationale for guiding future thromboprophylaxis. Results from both studies are expected during 2024. Various clinical studies are ongoing, randomizing between different antithrombotic strategies (Figure 3). In short, the ARMYDA-AMULET (ClinicalTrials.gov: NCT05554822) and APPENDAGE (ClinicalTrials.gov: NCT04796714) trials are comparing DAPT- versus SAPT-based strategies. Meanwhile, the ASPIRIN-LAAO (ClinicalTrials.gov: NCT03821883) trial compares continued ASA or complete discontinuation of all APT after an initial 6 months of DAPT following Watchman implantation. Similarly, trials comparing an antiplatelet-focused strategy to both full- and low-dose DOAC are ongoing. Of particular interest, the FADE-DRT (ClinicalTrials.gov: NCT04502017) trial implements a genetically tailored antithrombotic strategy with genotyping of clopidogrel responsiveness. 

While the trials presented above focus specifically on optimal thromboprophylaxis following LAAO, both the CATALYST (ClinicalTrials.gov: NCT04226547) and CHAMPION-AF (ClinicalTrials.gov: NCT04394546) trials look to compare LAAO to DOAC in DOAC-eligible patients [94]. With approximately 3000 participants enrolled in each study, these studies will constitute by far the largest randomized cohorts in LAAO research. 

In addition to the purely endocardial approach to LAA closure discussed in this review, surgical exclusion of the LAA can be achieved using either epicardial clipping or combined epicardial/endocardial ligation devices, such as the AtriClip and LARIAT systems. While more invasive than percutaneous LAAO, these approaches have been shown to be both safe and effective, while leaving no foreign material in contact with the circulation, thus avoiding the risk of DRT [95,96]. Including also patients having undergone LAA amputation, the randomized LAAOS III trial recently demonstrated a significantly reduced risk of thromboembolic events among (D)OAC-treated patients with a history of AF who underwent surgical occlusion of their LAA during on-pump cardiac surgery [97]. This additive effect of both pharmacological and surgical thromboembolic protection has led to recent guidelines recommending routine exclusion of the LAA during cardiac surgery among AF patients in addition to continued anticoagulation (level 1A) [98]. Nevertheless, the benefit of surgical LAA exclusion without continued anticoagulation is uncertain, as stated in the recent 2023 AHA guidelines on AF [98]. Building on these results of surgical exclusion, the planned LAAOS IV trial is a multicenter, prospective, open-labeled, randomized trial investigating the effects of Watchman FLX implantation as an add-on therapy for stroke prevention alongside DOAC.

With their inevitable impact on the position of LAAO, both the CATALYST, CHAMPION-AF, and LAAOS IV trials have the potential to significantly alter the LAAO population, therefore also adjusting the balance between thromboembolic and bleeding risk among LAAO patients, in turn affecting considerations when tailoring post-procedural antithrombotic therapy.

### 5.2. Next-Generation Devices 

With newer generation LAAO devices incoming, the need for aggressive post-procedural therapy may diminish. Animal data and recently presented human experience with the Watchman FLX Pro device indicate better facilitation of endothelialization and potentially a reduced risk of DRT by adding a fluoropolymer coating to the device fabric membrane [80]. Other devices, such as the Conformal and Laminar technologies, might further challenge the need for post-procedural treatment. With a very limited nitinol frame and a highly conformable and porous foam cup, the Conform Left Atrial Appendage Seal (CLAAS) design is meant to promote LAA sealing by improved adaption to the LA wall and increased tissue ingrowth [99,100]. In a preclinical canine study (*n* = 7), CLAAS achieved complete seal, no thrombus, and full neointimal coverage in all cases at 60 days [99]. Nevertheless, the limited published human data still include DAPT as discharge therapy [100,101].

The Laminar device promotes a completely novel approach to Left Atrial Appendage Exclusion (LAAX). Using a ball-and-lock mechanism, the device is advanced into the LAA where the ball is to engage with the LAA tissue followed by a rotational exclusion of the appendage. Subsequently, an atrial locking mechanism is deployed, leaving only a minimal surface area. Initial data from both canines (*n* = 9) and humans (*n* = 15) demonstrated complete sealing and no DRT during twelve months. The patients were treated with DOAC after LAA exclusion. 

### 5.3. Novel Factor XI Anticoagulants 

New targets of OAC therapies are being investigated with the development of factor XIa inhibitors [102]. Firstly, this approach appears promising following device implantation, as factor XIa is isolated to the intrinsic (contact activation) coagulation pathway, initiated through the exposed negatively charged surface of the device (Figure 1).

Secondly, in patients suffering from natural factor XI deficiency (hemophilia C), even very low factor XI plasma levels are associated with reduced risk of thrombosis, while only resulting in minor increased bleeding risk [103]. 

Despite the demonstrated safety of factor XIa inhibitors across phase 1 and 2 studies, with an apparent significant reduction in major bleeding, the recent premature termination of the OCEANIC-AF trial (ClinicalTrials.gov: NCT05643573) due to lack of efficacy of Asundexian compared to Apixaban has halted the enthusiasm for this approach [104,105,106,107,108].

## 6. Conclusions

LAAO still represents a relatively new treatment in AF, and much is still to be discovered about optimal post-procedural antithrombotics. Applied regimens vary greatly across studies, and reflect the heterogeneity of the LAAO population with variable bleeding and ischemic risk profiles. The current evidence seems in favor of a (low-dose) DOAC-alone or SAPT approach, given the high bleeding risk of current patient cohorts. A potential future shift in patient categories undergoing LAAO may alter the equilibrium between bleeding and thrombotic risk. Presently, a case-by-case tailoring of antithrombotic strategies should reflect the presence of DRT risk factors and patients’ bleeding and ischemic profiles. Several important randomized trials are ongoing, and may help further refine this individualized approach.

## Figures and Tables

**Table 1:** Identified patient and device-associated risk factors for device-related thrombosis.

	Specific Factors
**Patient factors**	Age [32] Female sex [32] History of stroke/TIA [33,34] High CHA_2_DS_2_-VASc [33] Non-paroxysmal AF [35,36] Hypercoagulable disorder [35] Chronic kidney disease [35] Echocardiographic parameters with LA low-flow Spontaneous echo contrast in the LA [37] Reduced left ventricular ejection fraction <40% [38,39]
**Procedural/device factors**	Deep device implant [35,37,40,41] Large LAAO device size [37,38] Exposed metal screw on the device surface Iatrogenic pericardial effusion [35]

**Table 2:** Overview of studies reporting specific outcomes in mixed AT cohorts.

AT-Mixed Studies						DRT *				Ischemic Stroke **				Major Bleeding **				CV Mortality **			
Study	Publ.	n	Device(s)	Random	FU (Months)	SAPT	DAPT	DOAC	VKA	SAPT	DAPT	DOAC	VKA	SAPT	DAPT	DOAC	VKA	SAPT	DAPT	DOAC	VKA
ACP registry [46]	2016	1047	ACP	No	13 (IQR; 6, 25)	4.4%				2.3%				2.1%							
EWOLUTION registry [47]	2017	893	WM	No	3	3.8%	3.1%	1.3%	0.8%	1.4%	0.5%	0.0%	0.0%	2.9%	1.6%	1.9%	2.0%				
Enomoto et al. [48]	2017	426	WM	No	4			1.0%	0.5%			0.0%	0.5%			0.9%	1.4%				
Fauchier et al. [34]	2018	469	ACP/AM/WM	No	13 (±13)	10.8%	1.2%	7.3%													
AMULET Registry [49]	2018	1078	AM	No	12	0.8%	1.6%	2.5%		2.9%				6.6%	8.4%	5.8%		8.3%			
ADRIFT Trial [31]	2020	105	ACP/AM/WM	Yes	3		6.1%	0.0%			0.0%	0.0%			21.2%	14.1%					
Patti et al. [50]	2020	610	ACP/AM/WM	No	12	1.4%	0.9%			1.8%	2.1%			2.9%	6.7%			6.0%	5.5%		
Faroux et al. [35]	2021	592	ACP/AM/WM	No	22 (IQR; 8, 38)		2.6%	0.0%			1.1%	1.7%			7.4%	3.2%					
Cepas-Guillén et al. [43]	2021	139	ACP/AM/WM/LB	No	3	7.7%	4.1%	0.0%		0.0%	1.4%	0.0%		0.0%	9.6%	0.0%					
AMULET IDE [18]	2021	1878	AM/WM(FLX)	No	18		3.3%	4.5%			1.7%	1.9%			10.6%	10.0%			3.1%	4.8%	
Freeman et al. [51]	2022	31.994	WM	No	6		3.3%	1.8%	1.8%		0.6%	0.6%	0.5%		3.3%	3.7%	4.4%				

* DRT rates are estimated based on patients with available follow-up imaging. ** Annual rates were chosen where possible for presentation of clinical events. Studies randomizing between LAAO and pharmacotherapy as well as between different AT regimens are marked as “random”. ACP, Amplatzer cardiac plug; AM, Amplatzer Amulet; AT, antithrombotic therapy; DAPT, dual antiplatelet therapy; DOAC, direct oral anticoagulation; DRT, device-related thrombosis; LAAO, left atrial appendage occlusion; LB, LAmbre; SAPT, single antiplatelet therapy; VKA, vitamin K antagonist; WM (FLX), Watchman (flx).

## Data Availability

Not applicable.

## Supplementary Materials

The following supporting information can be downloaded at: https://www.mdpi.com/article/10.3390/jcm13030803/s1, Table S1: Studies reporting on patients discharged on no antithrombotic therapy.
