# Supplementary material for: Left Atrial Appendage Occlusion and Post-procedural Antithrombotic Management
Source: J Clin Med. 2024 Jan 30;13(3):803. doi: 10.3390/jcm13030803 (PMC10856063; doi:10.3390/jcm13030803)
Supplement: Supplementary file 1 [file jcm-13-00803-s001.zip › jcm-2831975-supplementary.pdf]

Supplementary Table S1. Studies reporting on patients discharged on no antithrombotic therapy.

Study	Publ.	No therapy (%Total)	DRT	Ischemic stroke	Bleeding
ACP Registry [1]	2016	87 (8%)	-	-	-
EWOLUTION Registry [2]	2017	65 (6%)	2.3%%	0.0%%	1.6%
AMULET Registry [3]	2018	23 (2%)	0.0%	-	13%
Fauchier et al. [4]	2018	35 (8%)	22.2%	-	-
Darmon [5]	2020	22 (15%)	0.0%	0.0%	0.0%
Cepas-Guillén et al. [6]	2023	117 (9%)	-	-	-

1. Tzikas, A.; Shakir, S.; Gafoor, S.; Omran, H.; Berti, S.; Santoro, G.; Kefer, J.; Landmesser, U.; Nielsen-Kudsk, J.E.; Cruz-Gonzalez, I.; et al. Left atrial appendage occlusion for stroke prevention in atrial fibrillation: Multicentre experience with the AMPLATZER Cardiac Plug. *EuroInterv. J. EuroPCR Collab. Work. Group Interv. Cardiol. Eur. Soc. Cardiol.* **2016**, *11*, 1170–1179. https://doi.org/10.4244/eijy15m01_06.

2 Bergmann, M.W.; Betts, T.R.; Sievert, H.; Schmidt, B.; Pokushalov, E.; Kische, S.; Schmitz, T.; Meincke, F.; Stein, K.M.; Boersma, L.V.A.; et al. Safety and efficacy of early anticoagulation drug regimens after WATCHMAN left atrial appendage closure: Three-month data from the EWOLUTION prospective, multicentre, monitored international WATCHMAN LAA closure registry. *EuroInterv. J. EuroPCR Collab. Work. Group Interv. Cardiol. Eur. Soc. Cardiol.* **2017**, *13*, 877–884. https://doi.org/10.4244/eij-d-17-00042

3 Landmesser, U.; Tondo, C.; Camm, J.; Diener, H.C.; Paul, V.; Schmidt, B.; Settergren, M.; Teiger, E.; Nielsen-Kudsk, J.E.; Hildick-Smith, D. Left atrial appendage occlusion with the AMPLATZER Amulet device: One-year follow-up from the prospective global Amulet observational registry. *EuroInterv. J. EuroPCR Collab. Work. Group Interv. Cardiol. Eur. Soc. Cardiol.* **2018**, *14*, e590–e597. https://doi.org/10.4244/eij-d-18-00344.

4. Fauchier, L.; Cinaud, A.; Brigadeau, F.; Lepillier, A.; Pierre, B.; Abbey, S.; Fatemi, M.; Franceschi, F.; Guedeney, P.; Jacon, P.; et al. Device-Related Thrombosis after Percutaneous Left Atrial Appendage Occlusion for Atrial Fibrillation. *J. Am. Coll. Cardiol.* **2018**, *71*, 1528–1536. https://doi.org/10.1016/j.jacc.2018.01.076.

5. Darmon, A.; Couture, E.L.; Stein, G.; Cormier, B.; Chevalier, B.; Lefèvre, T.; Sanguineti, A.; Horvilleur, J.; Garot, P. Left Atrial Appendage Closure in Patients with Atrial Fibrillation at Very High Bleeding Risk without Postimplantation Antithrombotic Therapy. *J. Invasive Cardiol.* **2020**, *32*, 385–391

6. Cepas-Guillén, P.; Flores-Umanzor, E.; Leduc, N.; Bajoras, V.; Perrin, N.; Farjat-Pasos, J.; McInerney, A.; Lafond, A.; Millán, X.; Zendjebil, S.; et al. Impact of Device Implant Depth after Left Atrial Appendage Occlusion. *JACC Cardiovasc. Interv.* **2023**, *16*, 2139–2149. https://doi.org/10.1016/j.jcin.2023.05.045.
